# Measurement Properties of Instruments Used to Assess Diabetic Foot: An Umbrella Review

**DOI:** 10.1155/jdr/3322840

**Published:** 2026-06-11

**Authors:** Nagyeong Kim, Youngshin Song

**Affiliations:** ^1^ College of Nursing, Chungnam National University, Daejeon, Republic of Korea, cnu.ac.kr

**Keywords:** assessment instrument, COSMIN, diabetic foot, measurement properties, psychometrics, umbrella review

## Abstract

**Aims:**

Diabetic foot is a severe, chronic complication that contributes substantially to morbidity and mortality. Numerous instruments have been developed to support systematic foot evaluation; however, their measurement properties have not been sufficiently evaluated. This umbrella review is aimed to synthesize evidence from systematic reviews on diabetic foot assessment tools, evaluate their methodological quality, and compare the psychometric properties and clinical applicability of the identified tools.

**Materials and Methods:**

A systematic search of PubMed, EMBASE, the Cochrane Library, CINAHL, and Web of Science databases identified reviews published between 2010 and 2025. Two reviewers independently screened the studies, extracted data, and assessed methodological quality using the risk of bias in systematic reviews (ROBIS) and COnsensus‐based Standards for the selection of health Measurement INstruments (COSMIN) tools.

**Results:**

Eleven systematic reviews encompassing 24 instruments were analyzed. The ROBIS appraisal rated most reviews (73%) as having a high risk of bias. The instruments were grouped into four categories with frequent overlaps: screening, risk stratification, wound classification, and infection/healing monitoring. Evidence of reliability and construct validity was common, whereas measurement errors, responsiveness, and cross‐cultural validation were rarely assessed. Heterogeneity and methodological weaknesses limit robust conclusions.

**Conclusions:**

This umbrella review highlights the substantial gaps in the psychometric evaluation of assessment tools for diabetic foot, compounded by methodological flaws in supporting systematic reviews. Clinicians should exercise caution in tool selection, and future research should prioritize rigorous validation studies and high‐quality systematic reviews to establish standardized evidence‐based instruments.

## 1. Introduction

Diabetic foot is a serious chronic complication of diabetes, characterized by infection, ulceration, and/or destruction of deep tissues in the lower limbs, and is typically associated with peripheral neuropathy and varying degrees of peripheral arterial disease [1, 2]. This condition significantly contributes to morbidity and mortality among patients with diabetes, with diabetic foot ulcers (DFUs) being the most prevalent clinical manifestation [3]. The estimated global prevalence of DFUs is approximately 6.3%, posing substantial burdens on healthcare systems worldwide [4, 5].

DFUs are associated with high risks of serious complications, including soft tissue and bone infections, prolonged hospitalization, and nontraumatic lower limb amputations [6]. Up to 85% of diabetes‐related lower‐extremity amputations are preceded by a foot ulcer that progresses to severe infection or gangrene [7]. Given the high prevalence and clinical impact of diabetic foot, a clear understanding of its complications is essential, because they profoundly influence patient outcomes, quality of life, and healthcare costs [2, 8].

These complications predominantly arise from peripheral neuropathy and vascular insufficiency, which lead to tissue damage, delayed wound healing, and increased infection susceptibility [9, 10]. Neuropathic ulcerations are among the most common complications resulting from a loss of protective sensation caused by peripheral neuropathy [11]. Infections, including cellulitis, abscesses, and osteomyelitis, occur frequently because of impaired immunity and poor vascular supply [12, 13]. Charcot neuroarthropathy is a less common but serious complication, characterized by progressive joint dislocation and deformity due to autonomic neuropathy and repetitive trauma [14–16]. Lower extremity amputation may become unavoidable when tissue necrosis, severe infection, or nonhealing ulcers are present [17, 18]. Thus, if diabetic foot complications are not identified at an early stage, morbidity risk increases and individuals face substantial physical, social, psychological, and economic burdens, including higher mortality and loss of independence [19, 20].

Clinical guidelines emphasize routine foot examinations as the cornerstone of comprehensive diabetes care [21]. To support routine foot assessments, various diabetic foot screening tools have been developed to evaluate clinical parameters, such as sensory function, vascular status, skin condition, and foot structure [19]. The commonly used tools include the Inlow′s 60‐second Diabetic Foot Screen, International Working Group on the Diabetic Foot (IWGDF) risk stratification categories, perfusion, extent, depth/tissue loss, infection, sensation (PEDIS) classification system, and the site, ischemia, neuropathy, bacterial infection, and depth (SINBAD) score. These instruments rely on visual inspection, simple sensory tests, and structured clinical criteria to stratify patient risks and guide preventive and therapeutic decision‐making.

Previous studies and guidelines categorized diabetic foot assessment tools differently, reflecting diverse clinical purposes [19, 22]. For instance, some reviews grouped tools based on whether they assess neuropathy, ulceration risk, or wound healing, whereas guidelines, such as the IWGDF, distinguish between risk‐stratification systems, wound classification scores, and infection grading schemes [22]. Moreover, the characteristics and measurement properties of these tools were evaluated using various criteria. This heterogeneity highlights the lack of a unified framework for classifying diabetic foot measurement tools and judging their properties despite their widespread use in clinical practice and research.

Reflecting this challenge, various systematic reviews have aimed at synthesizing the characteristics, measurement properties, and clinical utility of assessment tools for diabetic foot from diverse perspectives. For example, systematic reviews investigated tools for evaluating diabetic foot wounds as well as instruments for risk classification, clinical diagnosis, and symptom screening. However, the measurement properties of these tools, such as their reliability, validity, responsiveness, and clinical applicability, are often inconsistently reported across studies, raising concerns regarding their optimal use in both clinical and research contexts [19, 23]. An umbrella review—a systematic review of existing systematic reviews—offers a rigorous and comprehensive approach for synthesizing current evidence on the measurement properties of diabetic foot assessment instruments [24, 25]. Therefore, an umbrella review is warranted to consolidate the fragmented evidence base, critically appraise methodological rigor, and provide unified evidence‐based guidance for both clinical practice and future research.

The present umbrella review is aimed at (a) synthesizing evidence from published systematic reviews on diabetic foot assessment tools, (b) evaluating the methodological quality and risk of bias of the included reviews, (c) classifying diabetic foot assessment tools according to their clinical purpose and compare their psychometric properties based on the COSMIN framework, and (d) identifying gaps in the current evidence base regarding the clinical applicability and psychometric evaluation of diabetic foot assessment tools.

## 2. Materials and Methods

### 2.1. Study Protocol and Registration

This umbrella review was conducted and reported in accordance with the preferred reporting items for systematic reviews and meta‐analyses–COnsensus‐based Standards for the selection of health Measurement INstruments (PRISMA‐COSMIN) guideline for outcome measurement instruments (OMIs) [26], which integrates the PRISMA [27] reporting standards with the COSMIN methodology [28, 29]. This review evaluated the measurement properties of instruments used for diabetic foot assessment. The protocol for this umbrella review is registered in the International Prospective Register of Systematic Reviews (PROSPERO CRD420251073116).

### 2.2. Search Strategy: Identification of Studies via Databases

We searched electronic databases, including PubMed, EMBASE, Cochrane Library, CINAHL, and Web of Science, for studies published between 2010 and June 19, 2025. For this review, the following population, exposure, comparison, and outcome (PECO) question was used: “Which instruments (E) for assessing diabetic foot conditions in individuals with diabetes (P) demonstrate acceptable measurement properties (O) and are applicable in clinical practice?” Each database was searched using keywords and medical subject heading (MESH) terms related to the keywords. The keywords and MESH terms were combined with “OR” and “AND” Boolean operators. To improve the precision and comprehensiveness of systematic review retrieval, we applied validated search filters developed by the InterTASC Information Specialists′ Subgroup (ISSG) [30]. In addition, we manually screened the reference lists of all included studies to identify potentially eligible studies that were not identified in the electronic search.

### 2.3. Inclusion and Exclusion Criteria

We included systematic reviews and meta‐analyses that examined the measurement properties of instruments for assessing diabetic foot. Eligible studies enrolled patients with diabetes mellitus, including those with suspected or confirmed diabetic foot disease (DFD). We also included studies in patients with diabetes whose medical history presented with symptoms characteristic of DFD, including DFU, diabetic peripheral neuropathy (DPN), distal symmetric polyneuropathy (DSPN), and amputation. No restrictions were placed on participant ethnicity, sex, or age. Studies using any measurement tool to measure diabetic foot outcomes were considered (exposure). Regarding the outcomes, studies reporting measurement properties (e.g., reliability, validity, and responsiveness) of diabetic foot assessment instruments were considered.

The exclusion criteria were as follows: studies not related to DFD (e.g., ulcer, amputation, neuropathy, ischemia, or infection); studies not involving diabetic foot‐related assessment tools (e.g., medical devices, predictive models, or instruments assessing quality of life or activities of daily living in patients with diabetic foot); studies with inappropriate methodologies, including primary studies (nonreviews), case studies, study protocols; and studies for which the full texts were unavailable.

### 2.4. Study Screening and Selection

The studies identified through the database searches were imported into reference management software (EndNote X9), and duplicate records were removed. The first author screened the titles and abstracts of the remaining studies to assess their relevance to the review topic. Potentially eligible studies were retained for full‐text review. Uncertainties or potential reasons for exclusion were documented separately during this phase. The corresponding author reviewed the study selection process and all included studies to ensure consistency and validity. Discrepancies at any stage were resolved through discussion between the first and corresponding author until consensus was reached.

### 2.5. Data Extraction and Coding Scheme

Two reviewers independently extracted the specified variables from each selected study. The following information was extracted from each review.

#### 2.5.1. Study Characteristics

The study characteristics are as follows: author(s), publication year, type of review, number of included studies, DFD instruments, review objectives, clinical setting (e.g., community, primary care, and hospital), psychometric properties reported, methodology or guidelines used (e.g., PRISMA and COSMIN), and specific focus of the review.

#### 2.5.2. Instrument Characteristics

Name of the tool, country of origin, number of tools identified in the included studies, target population (e.g., patients with DFU and diabetes), initial purpose (e.g., screening), number of items, scoring system, domains assessed, and user type (e.g., clinician‐administered and self‐reported). To ensure consistency and comparability of evidence across sources, only instruments reported in two or more reviews were retained for evaluation; those mentioned in a single review were excluded.

#### 2.5.3. Classification and Synthesis of Measurement Tool Properties

The tools addressed in the systematic reviews included in the analysis were classified according to previous guidelines and systematic reviews [19, 22, 31] for the purpose of measuring diabetic feet, such as screening, risk stratification, wound classification, and infection or healing monitoring. Using the COSMIN framework, we extracted data on psychometric properties, including internal consistency, reliability, measurement error, content validity, structural validity, hypothesis testing for construct validity, cross‐cultural validity, criterion validity, responsiveness, and interpretability. For tools evaluated in multiple reviews, we also considered the consistency and quality of the reported evidence (e.g., sufficient, insufficient, or indeterminate) [20].

### 2.6. Quality Appraisal of the Systematic Reviews

We assessed the quality of the included reviews using the Cochrane risk of bias in systematic reviews (ROBIS) tool. The ROBIS assessment covers four domains (study eligibility criteria; study identification; data collection; and study appraisal, synthesis, and findings) with judgments of low, high, or unclear risk. An overall ROBIS judgment was also recorded [32]. The ROBIS assessment was performed in two stages: (a) domain‐level assessment and (b) overall judgment, combining domain‐level ratings to determine an overall risk of bias classification (low, high, or unclear). We summarized the results in structured tables. Disagreements between reviewers were resolved through discussion or, when necessary, consultation with a third reviewer.

### 2.7. Classification and Properties of the Included Measurement Tools

We classified the tools for diabetic foot assessment into four categories according to the main clinical objectives presented in the guidelines and previous review studies [19, 22, 23].

1. Screening: Screening tools are aimed at identifying risk factors at an early stage, before ulceration develops, focusing on peripheral neuropathy, peripheral arterial disease, loss of protective sensation, and foot deformities. According to the IWGDF Practical Guidelines, regular screening provides a basis for preventive strategies and determines whether risk stratification is required.
2. Risk stratification: Risk stratification tools were designed to categorize patients into groups according to their risks for ulceration or amputation. These tools consider patient‐ and limb‐related characteristics, such as neuropathy, vascular disease, or a history of ulcers, and provide clinicians with criteria for adjusting follow‐up intervals and the intensity of preventive care.
3. Wound classification: Wound classification tools provide a structured assessment of existing DFUs by grading their severity and prognosis. Systems such as the Wagner classification, University of Texas classification, PEDIS, and SINBAD classify ulcers based on their depth, size, ischemia, infection, and tissue loss, thereby guiding treatment decisions and predicting clinical outcomes.
4. Infection/healing monitoring: Infection and healing monitoring tools evaluate the presence, extent, and progression of infection and are used to track healing; for example, by determining whether the ulcer has achieved complete epithelialization, monitoring the treatment response, and identifying the risk of recurrence.

The psychometric properties of each identified measurement tool were assessed according to the COSMIN taxonomy. Where applicable, we evaluated the following properties: content validity, structural validity, internal consistency, cross‐cultural validity, reliability, measurement error, criterion validity, hypotheses testing for construct validity, and responsiveness. Each property was rated as sufficient (+), insufficient (−), or indeterminate (?) depending on the quality and completeness of the supporting evidence. Interpretability was described separately because it is not classified as a psychometric property under the COSMIN framework. All evaluations were performed independently by two reviewers, and discrepancies were resolved through discussion or by a third reviewer when required. To ensure transparency and comparability across studies and instruments, the final ratings were summarized in structured tables.

## 3. Results

### 3.1. Literature Search

**Figure 1:**
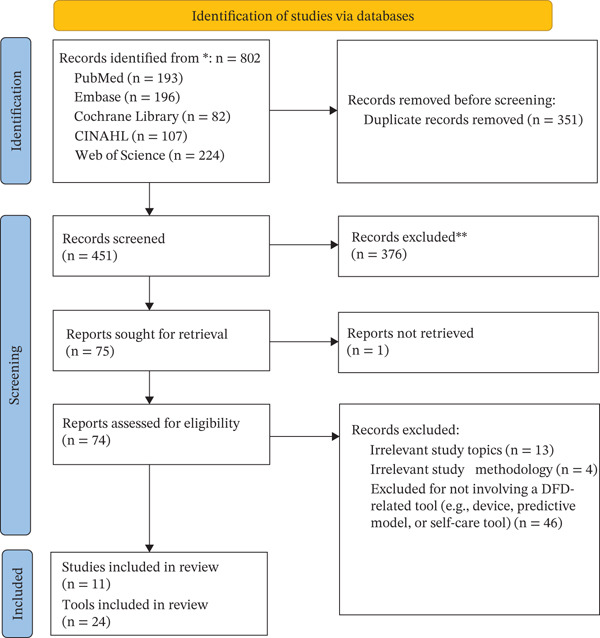
PRISMA flow diagram of the study screening and selection process. PRISMA, preferred reporting items for systematic reviews and meta‐analyses; DFD, diabetic foot disease.

A total of 802 studies were identified. After removing duplicates, completing the preliminary screening, and reviewing the full texts, the umbrella review ultimately included 11 studies. The study search and selection processes are presented in Figure 1.

### 3.2. Study Characteristics and Tool Outcomes

The main characteristics of the included studies are summarized in Table 1. Eleven systematic reviews published between 2010 and 2025 were included. Most (72.7%) were published within the last decade, reflecting growing interest in diabetic foot assessment. Among the included studies, three performed meta‐analyses (S3, S9, and S11) and eight (S1, S2, S4, S5, S6, S7, S8, and S10) comprised only systematic reviews. The number of studies included in the reviews varied between 6 (S5) and 149 (S10). These reviews examined 24 instruments for diabetic foot assessment used in diverse healthcare settings (Supplement 1). These tools ranged from simple screening instruments (e.g., United Kingdom Screening Test [UKST] and Basic Foot Screening Checklist [BFSC]) to comprehensive protocols for risk stratification (e.g., American Diabetes Association System [ADA] and IWGDF), wound classification (e.g., Wagner, PEDIS, and SINBAD), and infection or healing monitoring (e.g., Pressure Ulcer Scale for Healing [PUSH] and Bates–Jensen Wound Assessment Tool [BWAT]). Most studies were clinician‐administered and assessed domains such as neuropathy, peripheral arterial disease, ulcer depth and size, infection status, ischemia, and previous amputation. In addition, although some reviews used frameworks such as COSMIN to evaluate the measurement properties of the tool, reporting of validity, reliability, and responsiveness remained variable, underscoring the need for further standardization.

**Table 1:** General characteristics of included systematic reviews (*N* = 11).

Study	Author (year)	Review type	No. of included studies	Identified DFD instruments	Purpose of the review	Clinical setting (e.g., community, primary care, hospital)	Psychometric properties reported	Using method or guideline	Specific focus
1	Karthikesalingam et al. (2010)	SR	17	11 scoring systems +6 validation/comparative studies: Wagner, University of Texas (UT) system, DEPA, PEDIS, SINBAD, S(AD) SAD, NPUAP, and others.	To critically appraise the published literature of wound scoring systems for diabetic foot ulcers.	Mostly hospital and specialist diabetic foot clinics.	Validity and interrater reliability variably reported; limited data on responsiveness, measurement error.	The PRISMA guideline was followed for systematic review reporting. COSMIN was not used for comparison of validation studies.	Most diabetic foot ulcer scoring systems lack rigorous validation and exhibit limited reliability. Simplified tools such as SINBAD, DEPA, and DUSS show promise for clinical use, though external validation and interrater reliability data remain scarce. Among these, SINBAD is most commonly used, and simpler tools are generally recommended in clinical settings.
2	Monteiro‐Soares et al. (2011)	SR	13	5 risk classification systems: IWGDF risk classification system, Boyko′s tool, Leese system, and several ad hoc models.	To systematically identify and evaluate risk stratification systems for diabetic foot ulcer development. To assess the methodological quality and predictive validity of these systems.	Primary care and outpatient diabetes clinics.	Validity (predictive performance) evaluated in most tools; reliability and responsiveness not assessed.	STARD and STROBE guidelines were applied, with predictive metrics such as AUC, sensitivity, and specificity reported.	Risk stratification systems for diabetic foot ulcers are numerous, but generally show low methodological quality. Only a few systems, such as PODUS, Boyko, and IWGDF, have been externally validated and demonstrate acceptable predictive performance.
3	Monteiro‐Soares et al. (2014)	SR, MA	25	15 classification systems: Wagner, UT, DEPA, PEDIS, SINBAD, others.	To assess the ability of classification systems to predict lower extremity amputation (LEA) in patients with active DFUs.	Secondary/tertiary care (specialist foot clinics, hospitals).	Pooled predictive metrics (e.g., sensitivity, specificity); limited mention of reliability or responsiveness.	Partial use of STROBE/STARD and diagnostic metrics (AUC, sensitivity, specificity, LR) was reported.	The Wagner, S(AD)SAD, and University of Texas systems are the most frequently evaluated, yet overall evidence on their reliability and accuracy remains limited.
4	Ortega‐Avila et al. (2019)	SR	11	11 PROMs: DFS‐SF, FFI, FAAM, Q‐DFD, others.	To identify PROMs that are specific for DM affecting the foot and ankle and to evaluate their psychometric properties and methodological quality.	Community/outpatient	Internal consistency, construct validity assessed.	COSMIN and Terwee criteria were applied for quality and measurement assessment.	The Foot Health Status Questionnaire (FHSQ) demonstrates the highest psychometric quality among PROMs currently available for foot and ankle assessment in patients with diabetes.
5	Yoonhee and Youngshin (2019)	SR	6	6 DFU risk/assessment tools developed 2007–2016: foot assessment form, BFSC, SEWSS, UKST, a 60‐second tool, DFUAS.	To analyze the attributes and psychometric properties of newly developed diabetic foot complication assessment tools.	Primary care, community	Mentioned development/validation steps, limited detail.	No explicit guideline used; content analysis approach with some reporting of reliability and validity.	Only three tools reported reliability and validity, with most focusing on current status assessment rather than prediction. A variety of diabetic foot assessment tools were developed between 2007 and 2016 for diverse purposes; however, many lacked comprehensive psychometric validation. Further development and standardization—particularly for predictive and culturally adapted tools—are needed.
6	Fernández‐Torres et al. (2020)	SR	29	39 clinician assessment tools: PEDIS, SINBAD, DEPA, etc.	To carry out a systematic review of valid and reliable clinician assessment tools for measuring diabetic foot disease‐related variables and analyzing their psychometric properties.	Specialist clinical	Reliability, validity discussed; limited responsiveness.	COSMIN‐based evaluation included sensitivity, specificity, AUC, and interrater/intrarater reliability.	There is a general lack of comprehensive psychometric data for clinician‐administered assessment tools. However, several instruments demonstrate validity for specific purposes: UENS for diabetic neuropathy, QHRFF for ulceration risk, PEDIS and SINBAD for DFU assessment and amputation risk, and LUMT for ulcer measurement.
7	Pérez‐Panero et al. (2021)	SR	12	11 PROMs: DFSQ‐UMA, Q‐DFD, DFS‐SF, NeuroQol, etc.	To conduct a systematic review regarding the specific PROMs related to the evaluation of diabetic foot disease and to extract and analyze the values of their measurement properties.	Outpatient	Internal consistency, validity, responsiveness reported.	Measurement properties (validity, reliability, responsiveness) were assessed using the COSMIN checklist; PRISMA guidelines were followed.	Although most tools lack robust evidence, DFS‐SF and NeuroQoL show relatively strong properties. DFSQ‐UMA and Q‐DFD are recommended for comprehensive DFU assessment, though further validation is warranted.
8	Smet et al. (2021)	SR	27	14 chronic wound assessment tools (e.g., PUSH, BWAT)	(1) To systematically identify assessment tools for chronic wounds. (2) To examine their measurement properties and to summarize the data per assessment tool.	Hospital, nursing homes	COSMIN‐based appraisal; strong reliability and responsiveness.	Measurement properties were systematically assessed for each tool using the COSMIN Risk of Bias checklist, following PRISMA guidelines.	Most tools show limited psychometric quality. PUSH 3.0 and RESVECH 2.0 appear promising, though evidence is limited. Some validity and reliability findings are supported by moderate to high‐quality evidence.
9	Maldonado‐Valer et al. (2023)	SR, MA	36	IWGDF risk classification system	To determine the overall prevalence of diabetic foot at risk according to the International Working Group on the Diabetic Foot stratification.	Community, primary care	Prevalence‐focused review with limited psychometric detail; heterogeneity observed and subgroup analyses performed.	Subgroup prevalence and metaregression were performed; the review followed IWGDF, PRISMA, and was PROSPERO‐registered.	The global prevalence of foot at risk is 53.2%, with marked regional heterogeneity. The IWGDF classification is useful globally but requires careful contextual interpretation.
10	Monteiro‐Soares et al. (2024)	SR	149	28 Classification tools for DFU: UT, PEDIS, SINBAD	To assess published systems used to characterize ulcers in people with diabetes to determine which should be recommended to (a) aid communication between health professionals, (b) predict clinical outcome of individual ulcers, (c) characterize people with infection and/or peripheral arterial disease, and (d) audit to compare outcomes in different populations. This systematic review is part of the process of developing the 2023 guidelines to classify foot ulcers from the International Working Group on Diabetic Foot.	Specialist clinical	Validity, reliability, usability Updated assessment of previous systems	Validity, reliability, and clinical utility were assessed using PRISMA, IWGDF recommendations, and GRADE criteria.	Of 29 identified systems, six show potential for clinical use. Wagner, UT, and PEDIS were frequently reviewed, but most lack strong evidence. IWGDF offers context‐specific recommendations.
11	Welling et al. (2025)	SR/MA	96	13 Prognostic scores in PLAN framework: WIfI, GLASS, others	The 2019 Global Vascular Guidelines recommend risk assessment for evidence‐based revascularization based on the acronym PLAN: Patient risk, Limb severity and ANatomical complexity of disease. This meta‐analysis compares a multitude of prognostic tests within these categories.	Vascular surgery	AUC, sensitivity, specificity AUCs, calibration and validation presented	AUC, sensitivity, and specificity were assessed via Kaplan–Meier reconstruction and meta‐analysis. PRISMA, PROSPERO, GRADE, and MINORS were applied.	WIfI, GLASS, and GermanVasc are recommended under the PLAN concept for CLTI. Clinical use is advised only for tools with AUC ≥ 70*%*, though many lack sufficient reporting.

Abbreviations: AUC, area under the curve; COnsensus‐based Standards for the selection of health Measurement INstruments; DFD, diabetic foot disease; DFU, diabetic foot ulcer; IWGDF, International Working Group on the Diabetic Foot; MA, meta‐analysis; PROMs, patient‐reported outcome measures; PRISMA, preferred reporting items for systematic reviews and meta‐analyses; SR, systematic review; STARD, standards for reporting diagnostic accuracy studies; STROBE, strengthening the reporting of observational studies in epidemiology.

### 3.3. Quality Assessment of Included Systematic Reviews

We used the ROBIS system to assess the risk of bias at the systematic review level (Table 2). Overall, one review (9%) was judged to have a *low risk of bias*, two reviews (18%) had an *unclear risk*, and most (eight reviews; 73%) were classified as having a *high risk of bias*. At the domain level, the study eligibility criteria domain revealed high concerns in several reviews owing to the absence of prespecified protocols, insufficient justification for eligibility restrictions, and a lack of clarity regarding study selection procedures. In the identification and selection of studies domain, concerns were most often related to incomplete search strategies, exclusion of grey literature without justification, and inadequate documentation of the screening processes. For the data collection and study appraisal domains, high risk ratings were primarily attributed to the absence of duplicate data extraction, inconsistent use of validated risk of bias tools for primary studies, and insufficient reporting of data extraction procedures. In the synthesis and findings domain, the common limitations included a lack of sensitivity analyses, failure to explore publication bias, and limited integration of the primary study risk of bias assessments into the interpretation of the findings. The reviews rated as having *unclear risk* generally demonstrated stronger methodological safeguards, such as protocol registration, broader search coverage, and the use of established critical appraisal tools; however, they still lacked transparency in certain areas, particularly in reporting the operational definitions for eligibility criteria and conducting robustness checks of the synthesized results. The single *low-risk of bias* review adhered to best practices across all domains, including preregistration, comprehensive and unrestricted literature searches, duplicate screening, data extraction, application of validated appraisal tools, and explicit incorporation of risk‐of‐bias considerations.

**Table 2:** Quality assessment of the included studies according to Risk of Bias Assessment Tool for Systematic Reviews.

Study	Study eligibility criteria	Identification and selection of studies	Data collection and study appraisal	Synthesis and findings	Risk of bias in the review
1.Karthikesalingam et al. (2010)	High	High	High	High	High
2.Monteiro‐Soares et al. (2011)	High	High	High	High	High
3.Monteiro‐Soares et al. (2014)	High	High	High	High	High
4.Ortega‐Avila et al. (2019)	High	High	High	High	High
5.Yoonhee and Youngshin (2019)	High	High	High	High	High
6.Fernández‐Torres et al. (2020)	Unclear	Unclear	High	High	High
7.Pérez‐Panero et al. (2021)	Unclear	Unclear	High	High	High
8.Smet et al. (2021)	Unclear	Low	Low	Low	Unclear
9.Maldonado‐Valer et al. (2023)	Low	Low	Low	Low	Low
10.Monteiro‐Soares et al. (2024)	Unclear	Low	Unclear	Unclear	Unclear
11.Welling et al. (2025)	High	High	High	High	High

### 3.4. Psychometric Properties of Each Measurement Tool According to the Four Classifications

Table 3 presents the classification and summary of the psychometric properties measured using COSMIN. From the 11 systematic reviews, 24 tools for assessing diabetic foot were extracted and classified into four categories (screening tools, risk stratification instruments, wound classification tools, and infection or healing monitoring tools).

• Screening tools, including the UKST, BFSC, and Questionnaire for Diabetes‐Related Foot Disease (Q‐DFD), did not report measurement properties in most studies; therefore, most domains generally exhibited limited psychometric evidence, marked as “not reported,” “indeterminate,” or “insufficient.” The lack of psychometric evidence may be explained by their pragmatic design as brief screening instruments rather than rigorously validated measurement tools [19, 33]. These tools address key areas (e.g., peripheral neuropathy, vascular conditions, foot structure and deformation, and amputation history) and are used as primary screening tools for the early detection of high‐risk patients with diabetes [19, 33]. The advantages of these tools are that they can be easily applied in clinical settings without complex devices, and they are mainly used in primary care, outpatient clinics, and educational fields. However, rather than making a final diagnosis using the tool itself, high‐risk groups should be identified and undergo additional evaluations.
• Risk stratification instruments, including the Saint Elian Wound Score System (SEWSS), Diabetic Ulcer Severity Score (DUSS), and IWGDF, showed higher sufficiency ratings in construct validity but did not evaluate other domains such as internal consistency or measurement error. All these tools stratify the clinical risk of patients with diabetes, classify patients into low‐ to high‐risk groups, and use them for follow‐up, management, and treatment decisions. However, overall evidence supporting their measurement properties was limited. This gap reflects the fact that these instruments were developed primarily as clinical decision aids rather than psychometrically validated tools, that existing studies are often small, and that evaluation criteria and outcomes are heterogeneous [21, 23]. Several tools also overlap with other domains: DUSS includes wound severity features, WIfI integrates wound, ischemia, and infection to inform their potential for healing, and the Size (Area and Depth), Sepsis, Arteriopathy, and Denervation (S(AD)SAD) system links these tools to risk stratification, including ulcer size, depth, sepsis, and neuropathy, as well as wound classification and healing monitoring [19, 22, 34].
• Wound classification tools (e.g., SINBAD, PEDIS, and UT classification) showed some consistency in construct validity; however, structural validity and reliability data were often absent or inconsistent, as these tools were originally designed for clinical staging and communication rather than as rigorously validated measurement instruments [23]. Evidence for these systems remains limited because various tools (e.g., Wagner, PEDIS, and UT) have been developed and utilized for different purposes, such as prognosis prediction, treatment planning, and research standardization, making it difficult to accumulate consistent validation. Moreover, standardization studies are insufficient [23]. Furthermore, the heterogeneity of patients with diabetes, including vascular disease, neuropathy, and infection risk, as well as wide variations in wound location, size, and healing patterns, prevent the universal applicability of a single classification system [2, 23, 35, 36]. The inherent variability of wounds and subjectivity of clinical evaluation also restrict long‐term validation, thus relying on empirical expert judgment [37]. Despite these limitations, the tools mainly describe ulcer characteristics such as depth, infection, ischemia, and neuropathy, and are widely applied for clinical communication and treatment decisions [19, 23]. Several systems also integrate domains beyond classification: SINBAD incorporates ischemia, neuropathy, and infection, enabling its use in risk stratification; PEDIS includes infection and sensation, linking it to infection or healing monitoring; and the Curative Health Services (CHS) wound grade scale also captures wound status relevant for healing monitoring [19, 22, 38].
• Infection or healing monitoring tools, such as PUSH and DFUAS, showed stronger psychometric evidence overall, particularly in responsiveness and criterion validity, yet many lacked validation of internal consistency or cross‐cultural applicability. The limited evidence for these tools reflects the inherent variability of diabetic foot infections and healing, making it difficult to sensitively capture changes in long‐term follow‐up. Moreover, clinical practice still relies heavily on expert judgment [35, 37]. Additionally, the lack of a gold standard, patient heterogeneity, insufficient systematic validation, and multidisciplinary standardization have collectively hindered the accumulation of robust evidence on the reliability, validity, and responsiveness of these tools [37]. Despite these limitations, they are widely used in both clinical practice and research to quantify the wound status, healing trajectory, and infection burden. Some instruments also incorporate domains overlapping with classification systems: the depth of ulcer, extent of bacterial colonization, phase, etiology (DEPA) tool includes ulcer depth and extent, whereas the DFUAS and Photographic Wound Assessment Tool (PWAT) capture wound characteristics alongside healing monitoring, and BWAT evaluates tissue quality and infection features relevant to classification [19, 23].

**Table 3:** Classification and summary of measurement psychometric properties using COSMIN.

Classifications	COSMIN measurement property										
	Reliability			Validity					Responsiveness	Interpretability	Overall rating
	Internal consistency	Reliability	Measurement error	Content validity	Construct validity			Criterion validity	Responsiveness		
				Face validity	Structural validity	Hypotheses testing	Cross‐cultural validity				
**Screening Tools**											
UKST (the United Kingdom Screening Test)	NR	NR	NR	NR	NR	NR	NR	Insufficient	NR	NR	Insufficient
BFSC (Basic Foot Screening Checklist)	NR	Insufficient	NR	NR	NR	NR	NR	Insufficient	NR	NR	Insufficient
Q‐DFD (Questionnaire for Diabetes Related Foot Disease)	NR	Indeterminate (inconsistencies)	NR	Indeterminate	NR	Indeterminate (inconsistencies)	Sufficient	Indeterminate (inconsistencies)	NR	NR	NR or indeterminate
**Risk stratification tools**											
ADA (American Diabetes Association System)	NR	NR	NR	NR	NR	NR	NR	Sufficient	NR	NR	NR or indeterminate
SIGN (Scottish Intercollegiate Grouping Network System)	NR	NR	NR	NR	NR	NR	NR	Sufficient	NR	NR	NR or indeterminate
IWGDF (International Working Group on Diabetic Foot)	NR	Insufficient	NR	NR	NR	NR	NR	Sufficient	NR	NR	Insufficient
SEWSS (Saint Elian Wound Score System)	NR	Sufficient	NR	NR	NR	NR	NR	Sufficient	NR	NR	NR or indeterminate
DUSS (Diabetic Ulcer Severity Score) †	NR	NR	NR	NR	NR	NR	NR	Sufficient	NR	NR	NR or indeterminate
WIfI (Wound, Ischemia, and foot Infection) Classification †	NR	NR	NR	NR	NR	NR	NR	Sufficient	NR	NR	NR or indeterminate
DiaFoRa (Diabetic Foot Risk Assessment)	NR	NR	NR	NR	NR	NR	NR	Sufficient	NR	NR	NR or indeterminate
S(AD)SAD (Size [Area, and Depth], Sepsis, Arteriopathy, Denervation system) †	NR	NR	NR	NR	NR	NR	NR	Insufficient	NR	NR	Insufficient
**Wound Classification Tools**											
SINBAD (Site, Ischemia, Neuropathy, Bacterial Infection, and Depth) †	NR	Indeterminate (Inconsistencies)	NR	NR	NR	NR	NR	Sufficient	NR	NR	NR or indeterminate
(Meggitt‐) Wagner Classification	NR	Insufficient	NR	NR	NR	NR	NR	Indeterminate (inconsistencies)	NR	NR	insufficient
PEDIS (perfusion, extent, depth/tissue loss, infection, sensation classification) †	NR	Indeterminate (Inconsistencies)	NR	NR	NR	NR	NR	Sufficient	NR	NR	NR or indeterminate
UT (University of Texas) Classification	NR	Indeterminate (Inconsistencies)	NR	NR	NR	NR	NR	Sufficient	NR	NR	NR or indeterminate
CHS (Curative Health Services wound grade scale) †	NR	NR	NR	NR	NR	NR	NR	Indeterminate (inconsistencies)	NR	NR	NR or indeterminate
**Infection or Healing Monitoring Tools**											
DEPA (depth of ulcer, extent of bacterial colonization, phase, etiology) †	NR	NR	NR	NR	NR	NR	NR	Sufficient	NR	NR	NR or indeterminate
DFUAS (Diabetic Foot Ulcer Assessment Scale) †	NR	Sufficient	NR	Indeterminate	NR	Sufficient	NR	Sufficient	NR	NR	NR or indeterminate
PWAT (Photographic Wound Assessment Tool) †	NR	Indeterminate (Inconsistencies)	NR	NR	NR	NR	NR	Sufficient	NR	NR	NR or indeterminate
CSSC (Clinical Signs and Symptoms Checklist)	NR	Insufficient	NR	NR	NR	NR	NR	Insufficient	NR	NR	insufficient
PUSH (Pressure Ulcer Scale for Healing)	NR	Indeterminate	NR	Indeterminate	Indeterminate	Sufficient	NR	Sufficient	Sufficient	NR	NR or indeterminate
BWAT (Bates‐Jensen Wound Assessment Tool)	NR	Insufficient	NR	Indeterminate	Indeterminate	Indeterminate	NR	Sufficient	NR	NR	Insufficient
DMIST (Diabetic Foot Ulcer Assessment Tool)	NR	Sufficient	NR	NR	NR	Sufficient	NR	Sufficient	NR	NR	NR or indeterminate
DESIGN tool	NR	Indeterminate	NR	NR	NR	Sufficient	NR	Sufficient	NR	NR	NR or indeterminate

*Note:* +, sufficient; −, insufficient; ?, indeterminate; †, some tools may fit into more than one category.

Abbreviation: NR, not reported.

In summary, despite the differences in structure and development objectives, these tools show significant redundancy in the areas of evaluation and convergence in clinical applications. They are primarily utilized for risk classification, healing monitoring, and treatment decisions, and can ultimately be utilized in a similar clinical context. Such redundancy, together with the limited scope of the psychometric evaluation, was consistently reflected in subsequent COSMIN‐based appraisals of these instruments.

We evaluated the measurement properties of the 24 instruments used to assess diabetic foot within the COSMIN framework, with a focus on reliability, validity, responsiveness, and interpretability. Among the nine psychometric domains outlined by COSMIN, content validity, construct validity, and reliability were the most frequently evaluated. In contrast, measurement errors, cross‐cultural validity, and responsiveness were infrequently addressed or often not reported. Notably, no instrument demonstrated evidence across all nine measurement domains. A subset of tools, such as the DFUAS, Diabetic Foot Ulcer Assessment Tool (DMIST), and depth, exudate, size, inflammation/infection, granulation tissue, necrotic tissue (DESIGN) tools, showed relatively broader psychometric evaluation, particularly in reliability and construct validity domains. However, these tools lacked supporting data regarding measurement errors and cross‐cultural validity. Of all instruments reviewed, a substantial proportion had multiple domains with “not reported” (NR) or “indeterminate” (?) assessments, highlighting significant gaps in the psychometric evaluation of diabetic foot assessment tools.

## 4. Discussion

This umbrella review provides the first comprehensive synthesis of the measurement properties of instruments used for diabetic foot assessment by integrating evidence from systematic reviews published over the past 15 years. Across the 24 identified instruments, the findings revealed substantial heterogeneity in psychometric evaluation methods alongside widespread methodological limitations in the existing evidence base.

### 4.1. Principal Findings and Their Significance

The current landscape of diabetic foot assessment is marked by a paradox. Despite the proliferation of instruments designed to address the clinical imperative of systematic foot evaluation, few have undergone comprehensive psychometric scrutiny. Although the identified tools were grouped into four categories—screening, risk stratification, wound classification, and infection or healing monitoring–overlaps across domains were frequent, indicating that many instruments serve similar clinical purposes. For instance, wound classification systems such as SINBAD or PEDIS not only grade ulcer severity but also incorporate infection and ischemia status, thereby functioning simultaneously as infection monitoring tools [19, 23, 39]. Similarly, risk stratification frameworks often rely on variables such as ulcer history or peripheral arterial disease, which are also core parameters in wound classification systems, blurring the distinction between the two categories [23, 40]. This overlap underscores that these categories are not mutually exclusive but rather interdependent components of comprehensive diabetic foot care.

More importantly, the psychometric evidence underlying these instruments remains limited. Content validity, construct validity, and reliability were the most frequently examined properties. However, they were often evaluated selectively and inconsistently across instruments [19, 23]. Measurement error, a core property for interpreting changes at the individual patient level, was virtually absent from the reported evaluations, whereas responsiveness was examined in only a small subset of tools [23, 41]. Moreover, despite its critical importance in a condition with global prevalence, cross‐cultural validity has been neglected. Given the multifactorial nature of diabetic foot complications arising from neuropathy, vasculopathy, biomechanical alterations, and immune dysfunction, assessment instruments must demonstrate robust validity and reliability across these domains to ensure accurate risk stratification and informed clinical decision‐making. A limited number of instruments, including the DFUAS, DMIST, and DESIGN, have undergone relatively broader psychometric evaluations. Nevertheless, the absence of a thorough quantification of measurement error and responsiveness testing raises uncertainty regarding their capacity to detect clinically meaningful changes over time [19, 23, 41]. These findings mirror the broader pattern observed in other areas of chronic disease assessment, where limited forms of validation often substitute for a comprehensive psychometric evaluation [42]. Similar methodological gaps have been reported in related fields such as pressure ulcer and peripheral arterial disease assessment [43], underscoring the persistent challenge of developing instruments with robust and generalizable measurement properties.

In this context, it is crucial to consider whether the observed limitations reflect true methodological gaps or the inherent conceptual design of the instruments. The distinction between psychometric and clinimetric approaches is particularly relevant here. Although the COSMIN framework provides a structured methodology for evaluating measurement properties, not all instruments in this review were developed to measure latent constructs. Some tools—particularly those designed for risk stratification, classification, or clinical decision‐making—may be more appropriately characterized as clinimetric instruments. Although we utilized the COSMIN framework to ensure a consistent and systematic evaluation across heterogeneous instruments, certain properties, such as internal consistency or structural validity, may be less relevant for tools with a primarily clinical or composite structure. Consequently, the limited evidence in specific measurement domains should be interpreted with caution; these findings may reflect the conceptual intent and clinical function of the tools rather than mere methodological shortcomings. This underscores the necessity of balancing measurement rigor with clinical applicability when evaluating the quality of diabetic foot assessment tools.

### 4.2. Methodological Quality and Implications

Most of the included reviews displayed a moderate‐to‐high risk of bias. The COSMIN‐based assessment found no reviews with low risk, with approximately four out of five falling into the moderate range and the remainder showing high risk. The results of the ROBIS evaluation were more concerning, classifying nearly three quarters of the reviews as having a high risk of bias, with only one review meeting the low‐risk criteria. The common methodological weaknesses included missing or poorly described protocols, incomplete or language‐restricted search strategies, and the inconsistent application of validated appraisal frameworks. Several reviews lacked dual independent appraisals and, in many cases, the quality assessment findings were not integrated into the interpretation of the results. Such omissions increase the likelihood of overstating the clinical utility of certain instruments. The diversity of synthesis approaches also hinders meta‐analytical aggregation, limiting the ability to draw statistically robust conclusions regarding instrument performance.

These methodological limitations are not entirely academic. As systematic reviews underpin guideline development and policy formulation, flaws in review methodology carry the risk of embedding suboptimal tools into clinical pathways, with downstream implications for patient safety and healthcare resource allocation.

### 4.3. Clinical and Policy Implications

The gaps identified in psychometric validation have direct consequences for patient care. Instruments without demonstrated reliability may yield inconsistent results between assessors or across time, delaying the identification of high‐risk patients, or prompting inappropriate escalation of care. Tools lacking established responsiveness may fail to detect treatment effects or disease progression, thereby undermining individual‐level management and population‐level surveillance [19, 44]. From a policy perspective, the absence of robust measurement foundations impedes cost‐effectiveness analyses that rely on precise and valid outcome measures to inform adoption and resource allocation. Healthcare systems risk investing in tools with limited clinical value or failing to adopt those with genuine potential to improve their outcomes. The absence of cross‐cultural validation further limits the harmonization of international diabetes care standards, potentially perpetuating inequities in high‐burden populations [45, 46].

Several priorities emerge from this synthesis. First, comprehensive psychometric studies are urgently required for widely used instruments, particularly studies focusing on measurement errors and responsiveness. Such studies should apply rigorous methodological frameworks with adequate sample sizes and diverse clinical settings. Second, cross‐cultural validation is essential to ensure the equitable applicability of assessment tools across healthcare contexts, especially in low‐ and middle‐income countries where the diabetes burden is rapidly increasing. Third, direct comparative studies assessing multiple instruments in the same population could provide valuable evidence for informed tool selection. Finally, implementation research should investigate the barriers and facilitators to the adoption of psychometrically sound instruments in routine practice, ensuring that measurement quality translates into clinical utility.

### 4.4. Strengths and Limitations

This umbrella review applied dual appraisal frameworks (COSMIN and ROBIS), enabling the concurrent evaluation of instrument‐ and review‐level methodological quality. Restricting the inclusion of instruments to those assessed in multiple reviews enhanced reliability by reducing the influence of isolated findings. The focus on measurement properties rather than clinical outcomes alone ensured a targeted appraisal of psychometric robustness. However, this synthesis was constrained by the quality and completeness of the included systematic reviews. Variability in outcome definitions, reporting formats, and methodological approaches precluded meta‐analyses and necessitated narrative synthesis. Limiting the analysis to published systematic reviews may have led to the exclusion of recent primary studies that updated or refined the database. Differences in COSMIN applications further limit comparability across reviews. Although the four‐category classification was based on established guidelines and prior reviews, the categories were not entirely distinct. Overlaps between domains are unavoidable in infection and healing monitoring, particularly substantially intersecting wound classification. This conceptual overlap may limit the clarity of the framework and should be considered when interpreting the findings.

## 5. Conclusions

The results of this umbrella review highlight the substantial gaps in the psychometric evaluation of the tools used to assess diabetic foot, which are compounded by methodological shortcomings in supporting systematic reviews. Although certain tools show relatively strong evidence, none meet the full spectrum of the COSMIN criteria. The prevailing focus on content and construct validity, whereas neglecting measurement error, responsiveness, and cross‐cultural validity, is a serious limitation that may compromise clinical decision‐making and patient outcomes.

Clinicians should exercise caution in instrument selection, recognizing that apparent clinical utility does not guarantee robust measurement performance. Healthcare organizations should prioritize tools with the strongest available evidence, while acknowledging the current limitations. Coordinated, methodologically rigorous validation studies, and high‐quality systematic reviews are required to establish a robust, evidence‐based foundation for diabetic foot assessment that supports effective care delivery and efficient resource use.

## Author Contributions

Conceptualization: K.N. and S.Y.; methodology: K.N. and S.Y.; data curation: K.N. and S.Y.; formal analysis: K.N. and S.Y.; writing—original draft: K.N.; writing—review and editing: K.N. and S.Y.; supervision: S.Y.

## Funding

This work was supported by the National Research Foundation of Korea (NRF) under Grant No. RS‐2026‐25476867.

## Disclosure

The authors reviewed and approved all final content and take full responsibility for the manuscript.

## Conflicts of Interest

The authors declare no conflicts of interest.

## Supporting information


**Supporting Information** Additional supporting information can be found online in the Supporting Information section. Supplementary Material 1 Characteristics of diabetic foot measurement tools. Supplementary Material 2 The PRISMA‐COSMIN for OMIs 2024 statement checklist.

## Data Availability

The data that support the findings of this study are available from the corresponding author upon reasonable request. The umbrella review protocol is registered in PROSPERO (CRD420251073116).
