# Supplementary material for: Measurement Properties of Instruments Used to Assess Diabetic Foot: An Umbrella Review
Source: J Diabetes Res. 2026 Jun 11;2026:3322840. doi: 10.1155/jdr/3322840 (PMC13255150; doi:10.1155/jdr/3322840)
Supplement: Supplementary file 1 — Supporting Information Additional supporting information can be found online in the Supporting Information section. Supplementary Material 1 Characteristics of diabetic foot measurement tools. Supplementary Material 2 The PRISMA‐COSMIN for OMIs 2024 statement checklist. [file JDR-2026-3322840-s001.docx]

**Supplement 1. Characteristics of diabetic foot measurement tools**

|  | **DFD Instruments** | **Country of Origin** | **N reported in the studies** | **Initially designed for** | **Validated population** | **Designed for DFD** | **Scoring system** | **Number of items** | **Type (e.g., screening, risk prediction)** | **Domains Assessed** | **User Type (e.g., Clinician-administered, Patient self-report)** |
| --- | --- | --- | --- | --- | --- | --- | --- | --- | --- | --- | --- |
|  | **[Screening Tools]** |  |  |  |  |  |  |  |  |  |  |
| 1 | UKST (The United Kingdom Screening Test) | UK | (2/11) | Screening symptoms of diabetic peripheral neuropathy. | 120 with type 2 DM patients with and without peripheral neuropathy | Yes. Identifying patients at risk for diabetic foot disease (DFD). | Symptom score (maximum 9 points): 0–2: normal, 3–4: mild, 5–6: moderate, 7–9: severe. A symptom score of 5 or higher suggests neuropathy. / Symptom score (0–9), Sign score (0–10); higher scores indicate greater severity | Symptom: 5 items; Sign: 4 items | Screening | Symptom: abnormal sensation, site, timing, alleviating factor, night awakening; Sign: ankle reflex, pain, vibration, temperature | Includes 5 patient-reported symptom items (0–9) and 4 clinician-assessed sign items (0–10, Likert scale); symptoms are self-reported, signs are clinician-evaluated. |
| 2 | BFSC (Basic Foot Screening Checklist) | Canada | (2/11) | Quick clinical screening for high-risk diabetic foot | 1255 Adult patients with diabetes | Yes | 12-item checklist (yes/no for each risk factor) | 12 | Screening | Skin integrity, nail condition, sensation, pulses, foot deformity, ulcer presence, infection, footwear, etc. | Clinician-administered |
| 3 | Q-DFD (Questionnaire for Diabetes Related Foot Disease) | Australia | (2/11) | Screening diabetes-related foot disease | 31 patients with diabetes | Yes | 5 dimensions and 12 yes/no items (Dichotomous responses and open questions, "Yes" = 1, "No" = 0), total 0-12 | 12 | Screening, risk stratification | peripheral neuropathy, peripheral vasculopathy, foot ulceration, amputation and foot deformity | Primarily patient self-report; can be interview-administered |
|  | **[Risk Stratification Tools]** |  |  |  |  |  |  |  |  |  |  |
| 4 | ADA (American Diabetes Association) Diabetic Foot Risk Classification System | USA | (2/11) | Risk classification and management of diabetic foot | Adults with diabetes. Guidelines-based | Yes | Categorical classification into 4 risk levels (0–3) based on presence of neuropathy, deformity, PAD, and ulcer history. Risk 0: No neuropathy Risk 1: Neuropathy only Risk 2: Neuropathy + deformity and/or PAD Risk 3: History of foot ulcer or amputation | 4 main risk domains | Screening and risk stratification | Neuropathy, PAD, deformity, ulcer/amputation history | Clinician-administered |
| 5 | SIGN (Scottish Intercollegiate Grouping Network System) | Scotland | (4/11) | Risk stratification and management of diabetic foot | Adults with diabetes in Scotland, particularly in community settings | Yes | Categorical risk stratification into low, moderate, or high risk based on clinical foot findings | 4 | Screening and risk stratification | Neuropathy, PAD, deformity, ulcer/amputation history | Clinician-administered |
| 6 | IWGDF (International Working Group on Diabetic Foot) | International | (6/11) | Adults with diabetes in international, multi-center clinical settings | Adults with diabetes. Guidelines-based | Yes | 4 risk categories (0–3) Risk 0: No LOPS (Loss of Protective Sensation) or PAD Risk 1: LOPS or PAD Risk 2: LOPS + PAD and/or deformity Risk 3: History of ulcer or amputation | 4 | Screening and risk stratification | Neuropathy, PAD, deformity, ulcer/amputation history | Clinician-administered |
| 7 | SEWSS (Saint Elian Wound Score System) | Mexico | (4/11) | Ulcer severity and healing prediction | 1000+ Diabetic foot ulcer patients | Yes | 0-30 (higher = worse prognosis) | 10 | Wound severity and healing potential prediction | Wound size, depth, exudate, tissue, granulation | Clinician-administered |
| 8 | DUSS (Diabetic Ulcer Severity Score) † | Germany | (4/11) | Ulcer severity and healing prediction | 185 Adults with diabetes and active foot ulcers (mainly in inpatient or outpatient wound care settings) | Yes | Each of 4 items scored 0 or 1 → Total score ranges from 0 to 4 Higher score = more severe ulcer = higher risk of non-healing or amputation | 4 | Risk prediction (ulcer outcome) | Ulcer size, depth, sepsis, arteriopathy | Clinician-administered |
| 9 | WIFI (Wound, Ischemia, and foot Infection) Classification † | USA | (2/11) | Amputation risk and revascularization need | 1000+ Diabetic foot ulcer patients | Yes | 3 domains, each graded 0-3; combined staging | 3 | Risk stratification, severity | Wound extent, ischemia, infection | Clinician-administered |
| 10 | DIAFORA (Diabetic Foot Risk Assessment) | Italy | (2/11) | Risk assessment for diabetic foot complications | 1000+ Adults with diabetes in outpatient/primary care settings | Yes | Risk categories (low/moderate/high) | 8 | Risk prediction | Neuropathy, PAD, deformity, ulcer history, amputation | Clinician-administered |
| 11 | S(AD)SAD (Size, Area, Depth, Sepsis, Arteriopathy, Denervation system) † | France | (3/11) | Ulcer severity and outcome prediction | 100+ Diabetic foot ulcer patients | Yes | Each domain scored 0–3; total score 0–15 | 5 | Severity, outcome prediction | Size (Area), Depth, Sepsis, Arteriopathy, Denervation | Clinician-administered |
|  | **[Wound Classification Tools]** |  |  |  |  |  |  |  |  |  |  |
| 12 | SINBAD (Site, Ischaemia, Neuropathy, Bacterial Infection, and Depth) † | UK | (4/11) | Ulcer severity and outcome prediction | 1947 Diabetic foot ulcer patients | Yes | 0-6 points (1 per domain). Each of the 6 components is scored 0 or 1 → Total score: 0–6  Higher scores = greater severity and worse outcomes (healing/amputation) | 6 | Prognostic (severity & outcome prediction) | Site, ischaemia, neuropathy, bacterial infection, depth | Clinician-administered |
| 13 | Wagner Classification | USA | (4/11) | Ulcer grading/severity | 100+ Adults with diabetes and active foot ulcers, especially in surgical or wound care settings | Yes | Grading system from 0 to 5 based on ulcer depth and extent of tissue involvement. 0(intact skin), 1(superficial ulcer), 2(deep ulcer to tendon, bone or joint), 3(deep ulcer with abscess or osteomyelitis), 4(forefoot gangrene), 5(whole foot gangrene) | 6 grades (0–5) | Prognostic (ulcer severity grading) | Depth, gangrene, tissue involvement | Clinician-administered |
| 14 | PEDIS (Perfusion, Extent, Depth/Tissue Loss, Infection, Sensation Classification) † | International | (4/11) | Standardized assessment of diabetic foot ulcers, especially in clinical trials and outcome comparisons | 1000+ Diabetic foot ulcer patients | Yes | Each of the 5 domains is graded from 1 (mild) to 3 (severe). Ex) Perfusion Grade 2, Infection Grade 3. | 5 | Severity classification (descriptive and prognostic) | Perfusion, extent/size, depth/tissue loss, infection, sensation | Clinician-administered |
| 15 | UT (University of Texas) Classification | USA | (4/11) | Ulcer grading and staging | 194 Adults with diabetes and active foot ulcers, especially in wound care or surgical settings | Yes | Stage (A–D): Indicates presence of infection and/or ischemia  Grade (0–3): Indicates depth of ulcer  → Combined matrix (16 possible combinations: e.g., 1A, 2C, 3D, etc.) | 2 axes (depth × complication). 4 (grades) x 4 (stages). 16-square matrix (stage + grade) | Severity classification (descriptive & prognostic) | Depth, infection, ischemia | Clinician-administered |
| 16 | CHS (Curative Health Services wound grade scale) † | USA | (3/11) | Wound severity and healing prediction | 669 Diabetic foot ulcer patients | Yes | Wound Grades 0-4, based on depth and tissue involvement | 5 | Severity, healing prediction | Depth, infection, ischemia, necrosis, granulation | Clinician-administered |
|  | **[Infection or Healing Monitoring Tools]** |  |  |  |  |  |  |  |  |  |  |
| 17 | DEPA (Depth of Ulcer, Extent of Bacterial Colonization, Phase, Aetiology) † | India | (4/11) | Ulcer severity and healing prediction | 100 Diabetic foot ulcer patients | Yes | 1-3 points per domain, total 4-12 (higher = worse prognosis) | 4 | Prognostic (severity classification & wound healing prediction) | Depth, extent of bacterial colonization, phase, aetiology | Clinician-administered |
| 18 | DFUAS (Diabetic Foot Ulcer Assessment Scale) † | China | (4/11) | Ulcer severity and healing prediction | 180 Adults with diabetes and chronic foot ulcers, particularly in clinical wound care settings | Yes | 0-100 (multi-domain scale, higher = more severe) | 11 | Severity, healing prediction | Size, depth, exudate, infection, necrosis, granulation, etc. | Clinician-administered |
| 19 | PWAT (Photographic Wound Assessment Tool) † | Canada | (2/11) | Standardized photographic assessment of wounds, including diabetic foot ulcers | 100+ Adults with chronic wounds (including pressure ulcers, venous ulcers, diabetic foot ulcers) | Yes | 0-16 (4 domains, 0-4 each) | 4 | Wound assessment | Size, depth, edges, tissue type | Clinician-administered |
| 20 | CSSC (Clinical Signs and Symptoms Checklist) | USA/International | (2/11) | Clinical wound infection assessment | 100+ Chronic wound patients | Partially (infection detection, not full DFU severity) | risk categories (low/moderate/high) | 10 | Screening checklist (for infection risk, not wound severity) | Local signs of infection, exudate, periwound changes, healing trajectory | Clinician-administered |
| 21 | PUSH (Pressure Ulcer Scale for Healing) | USA | (3/11) | Pressure ulcer healing monitoring | 150+ Patients with chronic wounds, especially pressure ulcers | No | 0–17 (lower = better healing) | 3 | Healing monitoring | Wound surface area, Exudate amount, Tissue type | Clinician-administered |
| 22 | BWAT (Bates-Jensen Wound Assessment Tool) | USA | (2/11) | Assessment of chronic wounds (e.g. pressure ulcers, venous ulcers, diabetic foot ulcers) | 250+ Broad range of chronic wound patients, including those with DFUs | Yes (used in DFU monitoring and research) | 13–65 (higher = worse wound). 13 clinical items, each scored 1–5  Total score range: 13–65  Higher score = more severe wound | 13 | Wound severity and healing progress monitoring | Tissue type, exudate, size, surrounding skin, granulation, etc. | Clinician-administered |
| 23 | DMIST (Depth, Maceration, Infection/inflammation, Size, Tissue type) Score | Japan | (2/11) | Evaluating and predicting chronic wound healing, including diabetic foot ulcers (DFUs) | 100+ Patients with chronic wounds (especially DFUs, venous ulcers, pressure injuries) | Yes | 0–15 (higher = more severe) | 5 | Healing prognostic score | Depth, Maceration, Infection, Size, Tissue type | Clinician-administered |
| 24 | DESIGN-tool (Depth, Exudate, Size, Inflammation/Infection, Granulation tissue, Necrotic tissue) | Japan | (2/11) | Chronic wound evaluation, including pressure ulcers and diabetic foot ulcers | 1000+ Pressure ulcer patients | Yes | 0–66 (higher = more severe) | 7 | Severity, healing monitoring | Depth, Exudate, Size, Infection, Granulation, Necrosis, etc | Clinician-administered |

*†: Some tools may fit into more than one category.*

**Supplement 2. The PRISMA-COSMIN for OMIs 2024 statement checklist[26]**

| **Section and Topic** | **#** | **Checklist item^a^** | **Location** |
| --- | --- | --- | --- |
| TITLE | | | |
| Title | 1 | Identify the report as a systematic review and include as applicable the following (in any order): outcome domain of interest, population  of interest, name/type of OMIs of interest, and measurement properties of interest. | 1 |
| ABSTRACT | | | |
| OPEN SCIENCE | | | |
| Funding^b^ | 2.2 | Specify the primary source of funding for the review. | 19-20 |
| Registration | 2.3 | Provide the register name and registration number. | 19-20 |
| BACKGROUND | | | |
| Objectives | 2.4 | Provide an explicit statement of the main objective(s) or question(s) the review addresses. | 2 |
| METHODS | | | |
| Eligibility criteria | 2.5 | Specify the inclusion and exclusion criteria for the review. | 2 |
| Information sources | 2.6 | Specify the information sources (e.g., databases, registers) used to identify studies and the date when each was last searched. | 2 |
| Risk of bias | 2.7 | Specify the methods used to assess risk of bias in the included studies. | 2 |
| Measurement properties | 2.8 | Specify the methods used to rate the results of a measurement property. | 2 |
| Synthesis methods | 2.9 | Specify the methods used to present and synthesize results. | 2 |
| RESULTS | | | |
| Included studies | 2.10 | Give the total number of included OMIs and study reports. | 2 |
| Synthesis of results | 2.11 | Present the syntheses of results of OMIs, indicating the certainty of the evidence. | 2 |
| DISCUSSION | | | |
| Limitations of evidence | 2.12 | Provide a brief summary of the limitations of the evidence included in the review (e.g., study risk of bias, inconsistency, and imprecision). | 2 |
| Interpretation | 2.13 | Provide a general interpretation of the results and important implications. | 2 |
| PLAIN LANGUAGE SUMMARY | | | |
| Plain language summary | 3 | If allowed by the journal, provide a plain language summary with background information and key findings. | 2 |
| OPEN SCIENCE | | | |
| Registration and protocol | 4a | Provide registration information for the review, including register name and registration number, or state that the review was not registered. | 19-20 |
|  | 4b | Indicate where the review protocol can be accessed, or state that a protocol was not prepared. | 19-20 |
|  | 4c | Describe and explain any amendments to information provided at registration or in the protocol. | 19-20 |
| Support | 5 | Describe sources of financial or non-financial support for the review, and the role of the funders in the review. | 19-20 |
| Competing interests | 6 | Declare any competing interests of review authors. | 19-20 |
| Availability of data, code, and other materials | 7 | Report which of the following are publicly available and where they can be found: template data collection forms; data extracted from  included studies; data used for all analyses; analytic code; any other materials used in the review. | 19-20 |
| INTRODUCTION | | | |
| Rationale | 8 | Describe the rationale for the review in the context of existing knowledge. | 3-5 |
| Objectives | 9 | Provide an explicit statement of the objective(s) or question(s) the review addresses and include as applicable the following (in any  order): outcome domain of interest, population of interest, name/type of OMIs of interest, and measurement properties of interest. | 5 |
| METHODS | | | |
| Followed guidelines | 10 | Specify, with references, the methodology and/or guidelines used to conduct the systematic review. | 5 |
| Eligibility criteria | 11 | Specify the inclusion and exclusion criteria for the review. | 6 |
| Information sources | 12 | Specify all databases, registers, preprint servers, websites, organizations, reference lists and other sources searched or consulted to  identify studies. Specify the date when each source was last searched or consulted. | 5 |
| Search strategy | 13 | Present the full search strategies for all databases, registers, and websites, including any filters and limits used. | 5-6 |
| Selection process | 14 | Specify the methods used to decide whether a study met the inclusion criteria of the review, e.g., including how many reviewers  screened each record and each report retrieved, whether they worked independently, and if applicable, details of automation tools/AI  used in the process. | 6-7, 31 |
| Data collection process | 15 | Specify the methods used to collect data from reports, e.g., including how many reviewers collected data from each report, whether  they worked independently, any processes for obtaining or confirming data from study investigators, and if applicable, details of  automation tools/AI used in the process. | 6-7 |
| Data items | 16 | List and define which data were extracted (e.g., characteristics of study populations and OMIs, measurement properties’ results, and  aspects of feasibility and interpretability). Describe methods used to deal with any missing or unclear information. | 24-26, 32-35 |
| Study risk of bias assessment | 17 | Specify the methods used to assess risk of bias in the included studies, e.g., including details of the tool(s) used, how many reviewers  assessed each study and whether they worked independently, and if applicable, details of automation tools/AI used in the process. | 7-8 |
| Measurement properties | 18 | Specify the methods used to rate the results of a measurement property for each individual study and for the summarized or pooled  results, e.g., including how many reviewers rated each study and whether they worked independently. | 8-9 |
| Synthesis methods | 19a | Describe the processes used to decide which studies were eligible for each synthesis. | 7-9 |
|  | 19b | Describe any methods used to synthesize results. | 7-9 |
|  | 19c | If applicable, describe any methods used to explore possible causes of inconsistency among study results (e.g., subgroup analysis). | 7-9 |
|  | 19d | If applicable, describe any sensitivity analyses conducted to assess robustness of the synthesized results. | 7-9 |
| Certainty assessment | 20 | Describe any methods used to assess certainty (or confidence) in the body of evidence. | 7-9 |
| Formulating recommendations | 21 | If appropriate, describe any methods used to formulate recommendations regarding the suitability of OMIs for a particular use. | 7-9 |
| RESULTS | | | |
| Study selection | 22a | Describe the results of the search and selection process, from the number of records identified in the search to the number of study  reports included in the review, ideally using a flow diagram. If applicable, also report the final number of OMIs included and the number  of study reports relevant to each OMI. [T] | 9-10, 24-26, 31 |
|  | 22b | Cite study reports that might appear to meet the inclusion criteria, but which were excluded, and explain why they were excluded. | 24-26, 31 |
|  |  |  |  |
| OMI characteristics | 23a | Present characteristics of each included OMI, with appropriate references. [T] | 10, 32-35 |
|  | 23b | If applicable, present interpretability aspects for each included OMI. [T] | 10, 32-35 |
|  | 23c | If applicable, present feasibility aspects for each included OMI. [T] | 10, 32-35 |
| Study characteristics | 24 | Cite each included study report evaluating one or more measurement properties and present its characteristics. [T] | 10, 24-26 |
| Risk of bias in studies | 25 | Present assessments of risk of bias for each included study. [T] | 10-11, 27 |
| Results of individual studies | 26 | For all measurement properties, present for each study: (a) the reported result and (b) the rating against quality criteria, ideally using  structured tables or plots. [T] | 9-14, 28-30 |
| Results of syntheses | 27a | Present results of all syntheses conducted. For each measurement property of an OMI, present: (a) the summarized or pooled result and  (b) the overall rating against quality criteria. [T] | 11-14, 28-30 |
|  | 27b | If applicable, present results of all investigations of possible causes of inconsistency among study results. | 11-14, 28-30 |
|  | 27c | If applicable, present results of all sensitivity analyses conducted to assess the robustness of the synthesized results. | 11-14, 28-30 |
| Certainty of evidence | 28 | Present assessments of certainty (or confidence) in the body of evidence for each measurement property of an OMI assessed. [T] | 11-14, 28-30 |
| Recommendations | 29 | If appropriate, make recommendations for suitable OMIs for a particular use. | 11-14, 28-30 |
| DISCUSSION | | | |
| Discussion | 30a | Provide a general interpretation of the results in the context of other evidence. | 14-19 |
|  | 30b | Discuss any limitations of the evidence included in the review. | 14-19 |
|  | 30c | Discuss any limitations of the review processes used. | 14-19 |
|  | 30d | Discuss implications of the results for practice, policy, and future research. | 14-19 |
